# Folate and global health review series, part 4: syntheses on folate and autoimmune diseases and skeletal outcomes

**DOI:** 10.7189/jogh.16.04257

**Published:** 2026-07-03

**Authors:** Samantha Yoo, Azita Montazeri, Derrick Bennett, Yacong Bo, Peizhan Chen, Susan Duthie, Natalie Jensen, Atipatsa Kaminga, Jun-Shi Lai, Xue Li, Amanda J MacFarlane, Homero Martinez, Helene McNulty, Franco Momoli, Peter Mossey, Ron Munger, Rajendra Prasad Parajuli, Monique Potvin Kent, Michele Rubini, Marjanne Senekal, Lindsey Sikora, Alain Stintzi, Evropi Theodoratou, Hui Wang, Ann Yaktine, Julian Little

**Affiliations:** 1 School of Epidemiology and Public Health, Faculty of Medicine, University of Ottawa, Canada; 2 Nuffield Department of Population Health, University of Oxford, UK; 3 School of Public Health, Xinxiang Medical University, China; 4 Clinical Research Centre, Ruijin Hospital, Shanghai Jiao Tong University School of Medicine, Shanghai, China; 5 School of Pharmacy and Life Sciences, Robert Gordon University, Scotland, UK; 6 Department of Mathematics and Statistics, Faculty of Science, Technology and Innovation, Mzuzu University, Malawi; 7 Singapore Institute for Clinical Sciences, Agency for Science, Technology and Research, Singapore; 8 Centre for Population Health Sciences, University of Edinburgh, UK; 9 Nutrition Research Division, Health Canada, Canada; 10 Research and Development Unit, Nutrition International, Canada; 11 Nutrition Innovation Centre for Food and Health, School of Biomedical Sciences, Ulster University, Coleraine, Northern Ireland, UK; 12 School of Dentistry, University of Dundee, Scotland, UK; 13 Department of Nutrition, Dietetics, and Food Sciences, College of Agriculture and Applied Sciences, Utah State University, USA; 14 Herbert Wertheim School of Public Health and Human Longevity Science, University of California San Diego, USA; 15 Department of Neuroscience and Rehabilitation, University of Ferrara, Italy; 16 Department of Human Biology, Faculty of Health Sciences, University of Cape Town, South Africa; 17 Health Sciences Library, University of Ottawa, Canada; 18 School of Pharmacy, Faculty of Medicine, University of Ottawa, Canada; 19 Centre for Global Health, Usher Institute, College of Medicine and Veterinary Medicine, University of Edinburgh, UK; 20 School of Public Health, Faculty of Medicine, Shanghai Jiao Tong University, China; 21 Food and Nutrition Board, Health and Medicine Division, National Academies of Sciences, Engineering, and Medicine, USA

## Abstract

**Background:**

Autoimmune diseases and bone density loss (osteopenia and osteoporosis) are chronic conditions of complex aetiology that affect diverse populations. Folate may be associated with a higher risk of these disorders due to its essential role in one-carbon metabolism required for nucleotide synthesis, homocysteine metabolism, and methylation processes. However, the evidence on this association is inconclusive.

**Methods:**

We searched MEDLINE, Embase, CINAHL, the Cochrane Library, and the Database of Abstracts of Reviews of Effects from inception to February 2024 for systematic reviews and meta-analyses investigating the associations between folate exposure (dietary intake, supplementation, or blood concentrations) and any autoimmune diseases or skeletal outcomes. Pairs of researchers screened the retrieved syntheses, extracted the relevant data, assessed their risk of bias using the ROBIS tool, and evaluated the credibility of the evidence using predefined criteria.

**Results:**

We found 19 reviews reporting 25 unique associations: 15 on autoimmune diseases (multiple sclerosis, inflammatory bowel disease, psoriasis, human immunodeficiency virus, vitiligo, and systemic lupus erythematosus) and 10 on skeletal outcomes (fractures and bone mineral density loss). Most of the syntheses consisted of small-scale case-control studies. The risk of bias in the included syntheses was high. Owing to the small sample sizes, all the unique associations were assessed to be at a weak level of credibility.

**Conclusions:**

The evidence on the relationship between folate status and autoimmune diseases or skeletal outcomes was limited in breadth and depth. Most of the 25 unique associations were reported by a single synthesis comprising small-scale studies. Subgroup analyses or dose-response analyses were severely limited or unavailable. More well-powered, prospective studies investigating the relationships between folate and autoimmune and skeletal conditions are warranted.

**Registration:**

PROSPERO: CRD42021265041.

Autoimmune diseases include a wide range of disorders characterised by chronic inflammation and tissue and organ damage, which arise from the response to self-antigens [1], an immune-mediated attack on the body’s own organs [2]. Both the pathogenesis and the clinical manifestations of autoimmune diseases are diverse; a complex interaction between genetic variation and environmental factors, including infections [1,3,4], has been associated with disease onset, while individuals can experience acute or chronic symptoms, involving single or multiple organs, or systemic organ failure [3]. The prevalence of autoimmune diseases varies substantially by disease subtype [5,6], as a consensus on the definitions and criteria of diagnosis has yet to be reached [6]. Individuals with compromised autoimmunity are more susceptible to cardiovascular diseases [7–10].

Osteoporosis is also a chronic and multifactorial condition characterised by damage to bone tissue or a reduction in bone mineral density and deterioration of bone microarchitecture [11,12]. While old age, malnutrition, and some chronic diseases may contribute to lower bone mineral density and subsequently increase the risk of fractures, oxidative stress and deficiencies in certain nutrients with antioxidant properties are thought to play important roles, as well [13,14].

Folate’s functional role is in sustaining an one-carbon metabolism, involving the transfer and utilisation of one-carbon units in a network of pathways required for DNA and RNA biosynthesis, amino acid metabolism, and methylation processes [15]. The evidence on the relationship between folate status and the risk of autoimmune or skeletal conditions is rather limited, partly due to the evolving understanding of the aetiology and scope of these disorders and partly due to limitations in reporting folate exposure, with many analyses relying on reported dietary intake which typically lacks accuracy. Several meta-analyses have compared the circulating folate and vitamin B_12_ concentrations in individuals with various autoimmune diseases with those in healthy controls and reported inconclusive findings [9,10,16]. The literature on the association between folate and bone health is even more sparse. In this review, we sought to identify the available evidence using comprehensive search strategies and critically synthesise the knowledge on this topic.

## METHODS

We detailed the methodological framework used in this umbrella review in the first publication in this series [17]. Briefly, we searched MEDLINE, Embase, CINAHL, the Cochrane Library, and the Database of Abstracts of Reviews of Effects from inception to February 2024 for systematic reviews and meta-analyses investigating associations between folate intake or status and any autoimmune or skeletal outcome. We excluded syntheses examining homocysteine as a marker of folate exposure or those examining multivitamins or multiple nutrients without separate quantification of folate intake, as well as syntheses examining the effect of folic acid supplementation in reducing the side effects of methotrexate treatment in individuals being treated for rheumatoid arthritis or multiple sclerosis (because these did not investigate the relationship between folate exposure and development or treatment of autoimmune diseases). We set no restrictions on the study population or design of the component studies.

Pairs of reviewers (SY, AM, NJ) screened records in two stages (title/abstract and full-text) in Covidence (Veritas Health Innovation, Melbourne, Australia) and extracted the data, resolving discrepancies by consensus. Two researchers (AM, NJ) then assessed the methodological quality of the included syntheses using the ROBIS tool [18].

We categorised the evidence by type of exposure measure, outcome, and setting (population subgroups or geographical regions) to identify unique associations (unique exposure – unique outcome – unique setting). Any syntheses limited to specific population groups (*e.g.* age group, country, *etc*.) were treated as unique. For each category of unique associations, we examined the evidence for consistency in direction, magnitude, and statistical significance of the summary effects. If concordant, we selected the evidence with the largest sample size. If discordant, we selected the evidence based on the largest total sample size; the largest number of cases (for binary outcomes), recency of publication, and the highest methodological quality, as assessed by ROBIS. Finally, we evaluated the credibility of the selected evidence using predefined criteria (**Table 1**). Directional associations were graded as convincing, highly suggestive, suggestive, or weak, and null associations were graded as suggestive or weak. For unique associations that were assessed to be of a highly suggestive level of credibility, we recalculated the summary effects and 95% confidence intervals (CIs); predictive intervals to understand the dispersion of effect sizes [19]; heterogeneity between the studies using *I*^2^ and *P*-value; small study effects using Egger’s test of symmetry [20] with a significance threshold *P* < 0.10; and excessive significance [21] with a threshold *P* < 0.10, to the extent allowed by data availability.

**Table 1:** Criteria for credibility assessment

Category	Associations
**Directional associations**	
Convincing	
	With statistical significance of *P* < 10^−6^
	Based on ˃1000 cases (or ˃20 000 participants for continuous outcomes)
	For which largest component study reports a statistically significant result (*P* < 0.05) and has a 95 prediction interval that excludes the null
	Which do not have large heterogeneity (*I*^2^<50)
	Show no evidence of small study effects (*P* ˃ 0.10) or of excess significance bias (*P* ˃ 0.10)
Highly suggestive	With statistical significance of *P* < 10^−6^
	Based on ˃1000 cases (or ˃20 000 participants for continuous outcomes)
	For which largest component study reports a statistically significant result (*P* < 0.05)
Suggestive	With statistical significance of *P* < 0.01
	Based on ˃1000 cases (or ˃20 000 participants for continuous outcomes)
Weak	With statistical significance of *P* < 0.05
**Null associations**	
Suggestive	Based on ˃1000 cases (or ˃20 000 participants for continuous outcomes)
	Which do not have large heterogeneity (*I*^2^<50)
	With statistical significance of *P* > 0.10
Weak	With statistical significance of 0.05<*P* < 0.10

## RESULTS

### Overview of the search results

We identified 287 reviews for this umbrella review series [17], of which 19 (15 with meta-analyses) [9–11,16,22–36], investigated the relationships between folate intake/status and the risk of autoimmune diseases and skeletal conditions (Tables S2a and S2b in the **Online Supplementary Document**). Only six reviews (five meta-analyses) [11,31–33,35,36] reported on skeletal conditions. The evidence on autoimmune diseases primarily consisted of case-control studies (n = 85), while the evidence on skeletal conditions primarily consisted of cross-sectional studies (n = 50) and other observational studies (n = 33).

In the meta-analyses, a total of eight categories of outcomes were reported across 25 unique associations (Tables S3a and S3b in the **Online Supplementary Document**): multiple sclerosis, inflammatory bowel disease (any events, Crohn’s disease, ulcerative colitis), psoriasis, HIV, vitiligo, systemic lupus erythematosus, fracture, and bone mineral density/osteoporosis. Almost all of the identified unique associations were reported by a single review. Plasma/serum folate concentration was studied as the exposure measure in all unique associations. Risk estimates were reported as the mean differences in all but one association. Dietary folate intake was not investigated in any review.

### Risk of bias assessment

**Figure 1:**
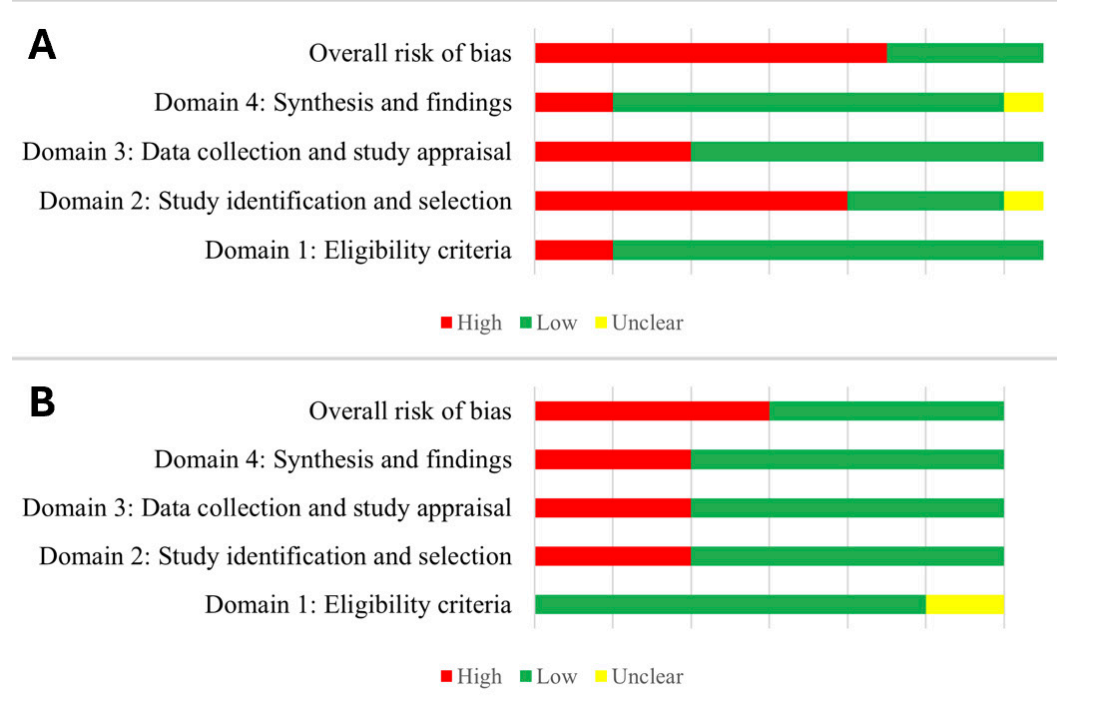
Risk of bias assessment of the included syntheses examining the relationship between folate intake/status and autoimmune diseases (**Panel A**) and folate intake/status and skeletal outcomes (**Panel B**).

While the overall risk of bias was high for syntheses examining both autoimmune diseases and skeletal outcomes (**Figure 1**, Panels A and B; and Tables S4a and S4b in the **Online Supplementary Document**), it varied substantially by domain. Most of the syntheses had a low risk of bias in the domains of defining study eligibility (83–85%), data abstraction and study appraisal (67–69%), and synthesis (67–77%). The risk of bias in the identification and selection of studies was high in approximately half of the syntheses reporting on folate status and autoimmune diseases (62%).

### Multiple sclerosis

Five meta-analyses examined the relationship between plasma/serum folate concentrations and the risk of multiple sclerosis by synthesising case-control studies [22–26]. All five syntheses reported null findings, albeit with varying magnitudes: the association was not significant in the general population (1161 participants, 1132 cases; standardised mean difference (SMD) = 0.07; 95% CI = −0.14, 0.28; *I*^2^ = 81) [24] or in the Chinese population (608 participants, 304 cases; SMD = −0.12; 95% CI = −0.29, 0.04; *I*^2^ = 45.3) [26].

The plasma/serum folate concentrations were not significantly different between individuals with relapsing multiple sclerosis (643 participants, 344 cases; SMD = −0.14; 95% CI = −0.42, 0.06; *I*^2^ = 53.6) and those with remitting multiple sclerosis (630 participants, 354 cases; SMD = −0.05; 95% CI = −0.51, 0.40; *I*^2^ = 76) [26].

### Inflammatory bowel disease

Mean serum folate concentration was significantly lower in individuals with inflammatory bowel disease compared to healthy controls (2522 participants, 1062 cases; SMD = −0.46 ng/mL; 95% CI = −0.64, −0.27; *I*^2^ = 74) [27]. This inverse association was consistent in the subgroup of studies conducted in Asia (1609 participants, 662 cases; SMD = −0.56 ng/mL; 95% CI = −0.86, −0.44; *I*^2^ = 64.3) and Europe (595 participants, 250 cases; SMD = −0.44 ng/mL; 95% CI = −0.62, −0.26; *I*^2^ = 3.1).

There was no significant difference in the mean serum folate concentration between individuals with Crohn’s disease and healthy controls (614 participants, 247 cases; SMD = −0.30 ng/mL; 95% CI = −0.63, 0.04; *I*^2^ = 79). However, individuals with ulcerative colitis presented significantly lower mean serum folate concentrations compared to healthy controls (1831 participants, 680 cases; SMD = −0.50 ng/mL; 95% CI = −0.71, −0.28; *I*^2^ = 63) [27].

A qualitative synthesis reported nine observational studies that measured red blood cell or serum folate concentrations in a paediatric population with inflammatory bowel disease with or without controls [28]. The studies were generally small in scale (sample sizes ranging from 41 to 154) and aggregated different subtypes of inflammatory bowel disease. Overall, the studies reported that 0–40 of the participants had low folate status (red blood cell count <5 ng/mL, cutoff for plasma concentration not provided).

### Psoriasis

Serum folate concentrations were significantly lower in individuals with psoriasis compared to healthy controls (1645 participants, 740 cases; SMD = −0.94; 95% CI = −1.49, −0.40; *I*^2^ = 95.6) [9]. The inverse association was consistently observed in a subgroup excluding individuals with psoriatic arthritis (1014 participants, 517 cases; SMD = −1.24; 95% CI = −2.05, −0.43; *I*^2^ = 96.7) or a subgroup of individuals with no prior exposure to methotrexate (769 participants, 393 cases; SMD = −2.13; 95% CI = −3.29, −0.97; *I*^2^ = 97.4).

### HIV

Significantly lower plasma folate concentrations were detected in individuals with HIV infection compared to healthy controls (505 participants, 255 cases; weighted mean difference = −2.74 ng/mL; 95% CI = −5.18, −0.29; *I*^2^ = 97) [29].

### Vitiligo

Serum folate concentrations were not significantly different between individuals with vitiligo and healthy controls (1050 participants, 672 cases; SMD = −0.24 ng/mL; 95% CI = −0.59, 0.11; *I*^2^ = 85.5) [16]. The associations remained null in a subgroup of individuals with progressive vitiligo (363 participants, 181 cases; SMD = −0.40 ng/mL; 95% CI = −1.55, 0.75; *I*^2^ = 95.7) or stable vitiligo (375 participants, 193 cases; SMD = −0.44 ng/mL; 95% CI = −1.41, 0.52; *I*^2^ = 94.3).

### Systemic lupus erythematosus

No significant differences in serum folate concentrations were observed between individuals with systemic lupus erythematosus and healthy controls (1166 participants, 532 cases; SMD = −0.28; 95% CI = −0.67, 0.12; *I*^2^ = 86.7) [10]. The null association remained in a subgroup excluding paediatric participants (452 participants, 302 cases; SMD = −0.36; 95% CI = −1.15, 0.44; *I*^2^ = 92.7).

### Alopecia areata

Three case-control studies examining the relationship between folate status and alopecia areata were synthesised narratively [30]. Two studies reported no difference in serum folate concentrations between individuals with alopecia areata and healthy controls, while one study reported that RBC folate concentrations were lower in individuals with alopecia universalis or alopecia totalis.

### Fracture

In a meta-analysis of prospective cohort studies of older individuals, severe folate deficiency (range not reported) was associated with an increased risk of fracture (7835 participants; hazard ratio = 1.46; 95% CI = 1.06, 2.02; *I*^2^ = 42.3). Low plasma folate status was not associated with an elevated risk of fracture (6470 participants; hazard ratio = 0.79; 95% CI = 0.56, 1.12; *I*^2^ = 0) [31].

### Bone mineral density and osteoporosis

A meta-analysis of cross-sectional studies reported no significant associations between plasma/serum folate concentrations and low bone mineral density in older women in the femoral neck (1037 participants; beta coefficient (*β*) = 0.00 nmol/L; 95% CI = −0.03, 0.03; *I*^2^ = 0), lumbar spine (1037 participants; *β* = 0.01 nmol/L; 95% CI = 0.00, 0.01; *I*^2^ = 0), or total hip (6366 participants; *β* = 0.00 nmol/L; 95% CI = 0.00, 0.01; *I*^2^ = 78.5) [11].

A meta-analysis of case-control studies reported no significant difference in serum folate concentrations between women with osteoporosis and healthy controls (649 participants, 288 cases; mean difference = −1.54; 95% CI = −3.33, 0.25; *I*^2^ = 94) [32]. In postmenopausal women, however, a synthesis of cross-sectional studies reported a significant difference in plasma/serum folate concentrations between women with osteoporosis and healthy controls (2793 participants, 735 cases; SMD = −1.16; 95% CI = −2.23, −0.08; *I*^2^ = 98.9) [33]. The inverse association remained significant in a subgroup of European studies (535 participants, 197 cases; SMD = −1.98; 95% CI = −3.70, −0.25; *I*^2^ = 98.4) and studies of serum samples (SMD = −1.27; 95% CI = −2.45, −0.10), but not in a subgroup of Asian studies (350 participants, 172 cases; SMD = −0.28; 95% CI = −0.74, 0.19; *I*^2^ = 76.2) [33].

### Credibility assessment

We assessed that all but one of the 25 unique associations had a weak level of credibility, predominantly due to small sample sizes (**Table 2**). We downgraded one association because it provided insufficient data.

**Table 2:** Identified unique associations between folate status and autoimmune/skeletal outcomes

Author (year)	Outcome, subgroup/setting	Primary study design	Exposure	Total number (number of cases	Metric	Summary estimate (95 CI)	Estimated *P*-value	*I*^2^ (%)	Credibility
Li *et al.* (2020) [24]	MS, general population	CC	Plasma	1161 (1132)	SMD	0.07 (−0.14, 0.28)	0.513	81	Weak
Pan *et al.* (2019) [26]	MS, Chinese population	CC	Plasma	608 (304)	SMD	−0.12 (−0.29, 0.04)	0.154	45.3	Weak
Pan *et al.* (2019) [26]	MS relapse, general population	CC	Plasma	643 (344)	SMD	−0.14 (−0.42, 0.06)	0.252	53.6	Weak
Pan *et al.* (2019) [26]	MS remission, general population	CC	Plasma	630 (354)	SMD	−0.05 (−0.51, 0.40)	0.829	76	Weak
Pan *et al.* (2017) [27]	IBD, general population	CC	Plasma	2522 (1062)	SMD	−0.46 ng/mL (−0.64, −0.27)	0.000	74	Weak
Pan *et al.* (2017) [27]	IBD, Asia	CC	Plasma	1609 (662)	SMD	−0.65 ng/mL (−0.86, −0.44)	<0.001	64.3	Weak
Pan *et al.* (2017) [27]	IBD, Europe	CC	Plasma	595 (250)	SMD	−0.44 ng/mL (−0.62, −0.26)	<0.001	3.1	Weak
Pan *et al.* (2017) [27]	Crohn’s Disease, general population	CC	Plasma	614 (247)	SMD	−0.30 ng/mL (−0.63, 0.04)	0.079	79	Weak
Pan *et al.* (2017) [27]	Ulcerative colitis, general population	CC	Plasma	1831 (680)	SMD	−0.50 ng/mL (−0.71, −0.28)	<0.001	63	Weak
Tsai *et al.* (2019b) [9]	Psoriasis, general population	CC	Plasma	1645 (740)	SMD	−0.94 (−1.49, −0.40)	<0.001	95.6	Weak
Tsai *et al.* (2019b) [9]	Psoriasis, excluding individuals with psoriatic arthritis	CC	Plasma	1014 (517)	SMD	−1.24 (−2.05, −0.43)	0.002	96.7	Weak
Tsai *et al.* (2019b) [9]	Psoriasis, individuals with no exposure to MTX	CC	Plasma	769 (393)	SMD	−2.13 (−3.29, −0.97)	<0.001	97.4	Weak
Deminice *et al.* (2015) [29]	HIV, general population	CS, PC, CC	Plasma	505 (255)	WMD	−2.74 ng/mL (−5.18, −0.29)	0.028	97	Weak
Tsai *et al.* (2019a) [16]	Vitiligo, general population	CC	Serum	1050 (672)	SMD	−0.24 ng/mL (−0.59, 0.11)	0.178	85.5	Weak
Tsai *et al.* (2019a) [16]	Vitiligo - progressive, general population	CC	Serum	363 (181)	SMD	−0.40 ng/mL (−1.55, 0.75)	0.495	95.7	Weak
Tsai *et al.* (2019a) [16]	Vitiligo – stable, general population	CC	Serum	375 (193)	SMD	−0.44 ng/mL (−1.41, 0.52)	0.374	94.3	Weak
Tsai *et al.* (2021) [10]	SLE, general population	CC	Plasma	1166 (532)	SMD	−0.28 (−0.67, 0.12)	0.092	86.7	Weak
Tsai *et al.* (2021) [10]	SLE, adults	CC	Plasma	452 (302)	SMD	−0.36 (−1.15, 0.44)	0.374	92.7	Weak
He *et al.* (2021) [31]	Fracture, older adults with severe folate deficiency	PC	Plasma	7835 (NR)	HR	1.46 (1.06, 2.02)	0.021	42.3	Weak
He *et al.* (2021) [31]	Fracture, older adults with low folate status	PC	Plasma	6470 (NR)	HR	0.79 (0.56, 1.12)	0.182	0	Weak (insufficient data)
Van Wijngaarden *et al.* (2013) [11]	BMD – femoral neck, older women	CS	Plasma	1037 (NR)	*β*	0.00 nmol/L (−0.03, 0.03)	1.000	0	Weak
Van Wijngaarden *et al.* (2013) [11]	BMD – lumbar spine, older women	CS	Plasma	1037 (NR)	*β*	0.01 nmol/L (0.00, 0.01)	<0.001	0	Weak
Van Wijngaarden *et al.* (2013) [11]	BMD – total hip, older women	CS	Plasma	6366 (NR)	*β*	0.00 nmol/L (0.00, 0.01)	1.000	78.5	Weak
Zhang *et al.* (2014) [32]	Osteoporosis, older women	CC	Plasma	649 (288)	MD	−1.54 (−3.33, 0.25)	0.091	94	Weak
Zhao *et al.* (2021) [33]	Postmenopausal osteoporosis	CS	Plasma/serum	2793 (735)	SMD	−1.16 (−2.23, −0.08)	0.034	98.9	Weak
Zhao *et al.* (2021) [33]	Postmenopausal osteoporosis, Asia	CS	Plasma/serum	350 (172)	SMD	−0.28 (−0.74, 0.19)	0.237	76.2	Weak
Zhao *et al.* (2021) [33]	Postmenopausal osteoporosis, Europe	CS	Plasma/serum	535 (197)	SMD	−1.98 (−3.70, −0.25)	0.024	98.4	Weak
Zhao *et al.* (2021) [33]	Postmenopausal osteoporosis	CS	Serum	NR (NR)	SMD	−1.27 (−2.45, −0.10)	0.034	NR	Weak

BMD – bone mineral density, CC – case-control study, CS – cross-sectional study, FA – folic acid, HR – hazard ratio, MA – meta-analysis, MD – mean difference, PC – prospective cohort, RCT – randomised controlled trial, RR – relative risk, SLE – systemic lupus erythematosus, SMD – standardised mean difference

## DISCUSSION

### Summary of findings

The existing evidence on the associations between folate intake/status and the risk of autoimmune diseases and skeletal outcomes is limited in breadth and depth. Among the 25 unique associations identified from 19 reviews, 15 reported on eight autoimmune diseases and 10 on fracture and bone mineral density. Most of these outcomes were examined by a single synthesis of case-control or cross-sectional studies. Subgroup analyses by geographic region were reported only for three outcome categories (multiple sclerosis, inflammatory bowel disease, postmenopausal osteoporosis), while dose-response relationships were not reported for any outcome. Many of the included syntheses reported high levels of heterogeneity potentially arising from differences in disease severity, use of medications, and other comorbidities. The overall risk of bias was high among the syntheses reporting on autoimmune diseases, particularly in the identification/selection of eligible evidence, and medium among the syntheses reporting on skeletal outcomes. All the evidence identified was found to be a weak level of credibility due to the small sample sizes.

Investigations of the relationship between folate status and autoimmune diseases appear to be an emerging area, with most of the identified evidence in this review having been published in the last 10 years. This trend may reflect the growing interest in the complex and unclear aetiology of these disorders, as well as their increasing global prevalence [4].

### Equity and global health in the evidence on autoimmune and skeletal outcomes

Most of the included syntheses consisted of studies conducted in both high-income countries (HICs) and low- and middle-income countries (LMICs), with two qualitative reviews pooling entirely from studies in LMICs [30,34]. Subgroup analyses by country income level would not have been feasible, likely owing to the small size of the samples pooled.

A global survey of autoimmune diseases revealed a substantial variation across countries in age-standardised, disability-adjusted life-years [4]. While the burden of autoimmune diseases from available evidence appears to be more impactful on HICs compared to LMICs, the authors if one paper noted potential under-detection of these complex disorders in LMICs due to their less-developed healthcare systems [4]. Some reports further suggest that certain autoimmune disorders such as systemic lupus erythematosus may clinically manifest more severely in individuals of Asian, African, and Hispanic ancestries [37–39].

The global disease burden of low bone mineral density has increased substantially in the last 30 years [40], with South Asia bearing the heaviest burden in terms of morbidity and mortality, and sub-Saharan Africa experiencing absolute increases in burden. In the context of limited access to or prioritisation of osteoporosis care in the LMICs [41], more research examining modifiable lifestyle risk factors for bone health is warranted.

We also note substantial variations in folate-related policy interventions across the countries. Although we did not identify reviews that directly investigated the impact of folic acid programmes on the distribution of autoimmune or skeletal outcomes at a population level, policies such as mandatory folic acid fortification can play an important role in improving folate status in the population [42].

### Biological plausibility of the evidence linking folate with autoimmune diseases and skeletal conditions

The relationship between folate status and the risk of autoimmune diseases or skeletal outcomes is not well understood, but likely relates to folate’s functional role in one-carbon metabolism and perturbations in this network as a consequence of deficient or low folate status [15]. Owing to its role in *de novo* nucleotide synthesis and cell proliferation, folate deficiency is hypothesised to be associated with a compromised immune system and low folate status may contribute to dysregulated proliferation of immune cells [43]. Folate is also involved in the production and maintenance of regulatory T cells in the gut and microbiome, which play an important role in immune homeostasis [44]. It is also involved in the methylation processes, which induce epigenetic modifications in the gut microbiota related to systemic immunity [45–47]. Folate deficiency may also induce genome instability [48], which may contribute to dysregulation of immune cells. Furthermore, folate is required for amino acid metabolism and low folate status leads to elevated homocysteine concentrations, in turn associated with disrupted collagen cross-linking in bone tissue, resulting in weakened bone strength [49–51], thereby increasing the risk of osteoporotic fractures. Evidence on the direct impact of high homocysteine concentration on fracture is scarce [52,53]. Elevated homocysteine may also be related to an increased risk of multiple sclerosis because of its impact on cell damage and macrophage activation [54] and myeline regeneration [55].

While more knowledge is needed to better understand the pathways potentially linking low folate status with autoimmune disorders, a recent Mendelian randomisation study [5] reported that a higher genetically-determined serum folate concentration was significantly associated with a reduced risk of vitiligo (odds ratio = 0.47; 95% CI = 0.32, 0.69).

### Limitations and next steps

This review has several limitations. First, the volume of evidence was insufficient; only some of the many different autoimmune diseases or skeletal outcomes were examined in the identified reviews. Most of the outcome categories were reported by a single review. Second, a predominant share of the syntheses consisted of case-control studies or cross-sectional studies, where the temporal relationship between exposure and outcome are difficult to ascertain. The retrospective nature of the primary studies was a concern particularly for inflammatory bowel disease, as affected individuals are often at risk of folate deficiency due to malabsorption. Prospective investigations are needed to answer whether low folate status is associated with the risk of developing inflammatory bowel disease. Third, the timing of blood sampling relative to the time of diagnosis was not clearly described and findings may be based on a mixture of chronic and newly diagnosed individuals with varying levels of severity. Relatedly, the variability of the assays or the clinical thresholds used in the component studies were not reported. Lastly, our search was limited to MEDLINE, Embase, CINHAL, the Cochrane Library, and DARE. We were unable to extend the search to CNKI and Wanfang, as described in the initial protocol, due to resource constraints. Considering the large volume of nutrition research conducted in China, omission of these databases may have introduced geographic and publication bias, particularly for autoimmune diseases with known regional variability. The potential impact of this bias is not substantial, at present, as research in this field is emerging; however, the Chinese databases should be included in future studies as evidence develops further.

Current knowledge on the relationship between folate intake/status and the risk of developing autoimmune or skeletal conditions could benefit from more well-designed, prospective investigations that examine potentially differential risks by age, sex, and other underlying risk factors. More specifically, longitudinal investigations adjusting for exposures to folic acid fortification, comorbidities of the study population, and standardised assay methods should be considered in the future. Genotypes known to be associated with folate status can also be examined to further strengthen triangulation of the epidemiological evidence.

## CONCLUSIONS

We systematically synthesised the evidence between folate intake/status and autoimmune and skeletal outcomes. The evidence identified was limited in breadth and depth. Most of the 25 unique associations were reported by a single synthesis comprising small-scale studies. Subgroup analyses or dose-response analyses were severely limited or unavailable. We did not identify any evidence at a suggestive level of credibility or higher. More well-powered, prospective studies investigating the relationships between folate and autoimmune and skeletal conditions are warranted.

## Additional material


Online Supplementary Document
