# Supplementary material for: Folate and global health review series, part 4: syntheses on folate and autoimmune diseases and skeletal outcomes
Source: J Glob Health. 2026 Jul 3;16:04257. doi: 10.7189/jogh.16.04257 (PMC13329937; doi:10.7189/jogh.16.04257)
Supplement: Online Supplementary Document [file jogh-16-04257-s001.pdf]

Supplement to: Yoo S, Montazeri A, Bennett D, Bo Y, Chen P, Duthie S, Jensen N, Kaminga A, Lai J, Li X, MacFarlane A, Martinez H, McNulty H, Momoli F, Mossey P, Munger R, Parajuli RP, Kent M, Rubini M, Senekal M, Sikora L, Stintzi A, Theodoratou E, Wang H, Yaktine A, Little J. Folate and global health review series, part 4: syntheses on folate and autoimmune diseases and skeletal outcomes. J Glob Health. 2026;16:04257.

Table S1. Full search strategy

**Medline**	
1.	exp Folic Acid/
2.	((vitamin* or vit or co?enzym*) adj2 (b9 or b 9 or m)).tw,kw.
3.	(folate or folic acid or folacin or folvite or pteroylglutamic acid or acfol or acifolic or acido folico or filicine or folacid or folart or folavit or folavite or foldivie or foliamin or folicid or folicet or folina or folinsyre or folitab or folium acid or folivit or folsan or folsau or folveriam or folvite or ingafol or gravi-fol or lafol or lexpec or megafol or neocepri or pteroyl glutamate or pteroyl monoglutamate or pteroyl monoglutamic acid or rubiefol or unifol).tw,kw.
4.	or/1-3
5.	diet/ or eating/ or drinking/
6.	((calorie or calories or caloric or diet* or feed* or food* or macronutrient* or micronutrient* or nutrient* or nutritional) adj2 (intake or intakes)).tw,kw.
7.	ingest*.tw,kw.
8.	Dietary Supplements/
9.	((diet* or food or herbal) adj2 supplement*).tw,kw.
10.	(neutraceutical* or nutracentical*).tw,kw.
11.	Food Preferences/
12.	exp Nutrition Therapy/
13.	Foods, Fortified/
14.	((fortified or enriched or supplement*) adj2 food*).tw,kw.
15.	Nutritional Status/
16.	((nutrition* or food*) adj2 status*).tw,kw.
17.	exp Homocysteine/
18.	exp Plasma/
19.	Erythrocytes/
20.	exp Serum/
21.	((biologic* or clinical or biochemical or serum or immun*) adj2 (marker or markers)).tw,kf.
22.	((end point or end points or endpoint or endpoints) adj surrogate).tw,kf.
23.	homocysteine.tw,kf.
24.	or/5-23
25.	4 and 24
26.	meta-analysis/ or "systematic review"/
27.	Systematic Reviews as Topic/
28.	(systematic adj2 review*).tw,kw.
29.	systematic review.pt.
30.	(meta analys* or metaanalys*).tw,kw.
31.	meta analysis.pt.
32.	(((systematic or state-of-the-art or scoping or literature or umbrella) adj (review* or overview* or assessment*)) or "review* of reviews" or meta-analy* or metaanaly* or ((systematic or evidence) adj1 assess*) or "research evidence" or metasynthe* or meta-synthe*).tw.
33.	or/26-32
34.	25 and 33
**Embase**	
1.	folic acid/

2. ((vitamin* or vit or co?enzym*) adj2 (b9 or b 9 or m)).tw,kw.
3. (folate or folic acid or folacin or folvite or pteroylglutamic acid or acfol or acifolic or acido folico or filicine or folacid or folart or folavit or folavite or foldivie or foliamin or folicid or folicet or folina or folinsyre or folitab or folium acid or folivit or folsan or folsau or folveriam or folvite or ingafol or gravi-fol or lafol or lexpec or megafol or neocepri or pteroyl glutamate or pteroyl monoglutamate or pteroyl monoglutamic acid or rubiefol or unifol).tw,kw.
4. or/1-3
5. diet/
6. exp food intake/
7. ((calorie or calories or caloric or diet* or feed* or food* or macronutrient* or micronutrient* or nutrient* or nutritional) adj2 (intake or intakes)).tw,kw.
8. ingest*.tw,kw.
9. ingestion/
10. diet supplementation/ or dietary supplement/
11. ((diet* or food or herbal) adj2 supplement*).tw,kw.
12. (neutraceutical* or nutracentical*).tw,kw.
13. food preference/
14. exp diet therapy/
15. fortified food/
16. ((fortified or enriched or supplement*) adj2 food*).tw,kw.
17. nutritional status/
18. ((nutrition* or food*) adj2 status*).tw,kw.
19. homocysteine/
20. exp plasma/
21. erythrocyte/
22. exp serum/
23. ((biologic* or clinical or biochemical or serum or immun*) adj2 (marker or markers)).tw,kf.
24. ((end point or end points or endpoint or endpoints) adj surrogate).tw,kf.
25. homocysteine.tw,kf.
26. or/5-25
27. meta-analysis/ or systematic review/ or systematic reviews as topic/ or meta-analysis as topic/ or "meta analysis (topic)"/ or "systematic review (topic)"/ or exp technology assessment, biomedical/ or network meta-analysis/
28. ((systematic* adj3 (review* or overview*)) or (methodologic* adj3 (review* or overview*))).ti,ab,kf,kw.
29. ((quantitative adj3 (review* or overview* or synthes*)) or (research adj3 (integrati* or overview*))).ti,ab,kf,kw.
30. umbrella review*.ti,ab,kf,kw.
31. ((integrative adj3 (review* or overview*)) or (collaborative adj3 (review* or overview*)) or (pool* adj3 analy*)).ti,ab,kf,kw.
32. or/27-31
33. 4 and 26 and 32

CDSR

1. ((vitamin* or vit or co?enzym*) adj2 (b9 or b 9 or m)).tw,kw.
2. (folate or folic acid or folacin or folvite or pteroylglutamic acid or acfol or acifolic or acido folico or filicine or folacid or folart or folavit or folavite or foldivie or foliamin or folicid or folicet or folina or folinsyre or folitab or folium acid or folivit or folsan or folsau or folveriam or folvite or ingafol or gravi-fol or lafol or lexpec or megafol or neocepri or pteroyl glutamate or pteroyl monoglutamate or pteroyl monoglutamic acid or rubiefol or unifol).tw,kw.
3. or/1-2
4. ((calorie or calories or caloric or diet* or feed* or food* or macronutrient* or micronutrient* or nutrient* or nutritional) adj2 (intake or intakes)).tw,kw.

5. ingest*.tw,kw.
6. ((diet* or food or herbal) adj2 supplement*).tw,kw.
7. (neutraceutical* or nutracentical*).tw,kw.
8. ((fortified or enriched or supplement*) adj2 food*).tw,kw.
9. ((nutrition* or food*) adj2 status*).tw,kw.
10. ((biologic* or clinical or biochemical or serum or immun*) adj2 (marker or markers)).tw,kw.
11. ((end point or end points or endpoint or endpoints) adj surrogate).tw,kw.
12. homocysteine.tw,kw.
13. or/4-12
14. 3 and 13

DARE

1. ((vitamin* or vit or co?enzym*) adj2 (b9 or b 9 or m)).tw,kw.
2. (folate or folic acid or folacin or folvite or pteroylglutamic acid or acfol or acifolic or acido folico or filicine or folacid or folart or folavit or folavite or foldivie or foliamin or folicid or folicet or folina or folinsyre or folitab or folium acid or folivit or folsan or folsau or folveriam or folvite or ingafol or gravi-fol or lafol or lexpec or megafol or neocepri or pteroyl glutamate or pteroyl monoglutamate or pteroyl monoglutamic acid or rubiefol or unifol).tw,kw.
3. or/1-2
4. ((calorie or calories or caloric or diet* or feed* or food* or macronutrient* or micronutrient* or nutrient* or nutritional) adj2 (intake or intakes)).tw,kw.
5. ingest*.tw,kw.
6. ((diet* or food or herbal) adj2 supplement*).tw,kw.
7. (neutraceutical* or nutracentical*).tw,kw.
8. ((fortified or enriched or supplement*) adj2 food*).tw,kw.
9. ((nutrition* or food*) adj2 status*).tw,kw.
10. ((biologic* or clinical or biochemical or serum or immun*) adj2 (marker or markers)).tw,kw.
11. ((end point or end points or endpoint or endpoints) adj surrogate).tw,kw.
12. homocysteine.tw,kw.
13. or/4-12
14. 3 and 13

CINAHL

(MH "Diet+")
(MH "Eating")
(MH "Fluid Intake") OR (MH "Food Intake+") OR (MH "Dietary Reference Intakes")
(MH "Dietary Supplements+") OR (MH "Food, Fortified") OR (MH "Nutrients+")
(MH "Nutritional Status")
(MH "Food Preferences")
(MH "Diet Therapy+")
((calorie or calories or caloric or diet* or feed* or food* or macronutrient* or micronutrient* or nutrient* or nutritional) N2 (intake or intakes))
ingest*
((diet* or food or herbal) N2 supplement)
(neutraceutical* or nutracentical*)
((fortified or enriched or supplement*) N2 food*)
((nutrition* or food*) N2 status*)
(MH "Homocysteine")
(MH "Plasma+") OR (MH "Serum")
(MH "Erythrocytes+")
((biologic* or clinical or biochemical or serum or immun*) N2 (marker or markers))
((end point or end points or endpoint or endpoints) N1 surrogate)
homocysteine

S1 OR S2 OR S3 OR S4 OR S5 OR S6 OR S7 OR S8 OR S9 OR S10 OR S11 OR S12 OR S13 OR S14 OR S15 OR S16 OR S17 OR S18 OR S19
(MH "Folic Acid+")
((vitamin* or vit or co?enzym*) N2 (b9 or b 9 or m))
(folate or folic acid or folacin or folvite or pteroylglutamic acid or acfol or acifolic or acido folico or filicine or folacid or folart or folavit or folavite or foldivie or foliamin or folicid or folicet or folina or folinsyre or folitab or folium acid or folivit or folsan or folsau or folveriam or folvite or ingafol or gravi-fol or lafol or lexpec or megafol or neocepri or pteroyl glutamate or pteroyl monoglutamate or pteroyl monoglutamic acid or rubiefol or unifol)
S21 OR S22 OR S23
S20 AND S24
(MH "meta analysis" OR MH "systematic review" OR MH "Technology, Medical/EV" OR PT "systematic review" OR PT "meta analysis" OR (((TI systematic* OR AB systematic*) N3 ((TI review* OR AB review*) OR (TI overview* OR AB overview*))) OR ((TI methodologic* OR AB methodologic*) N3 ((TI review* OR AB review*) OR (TI overview* OR AB overview*)))) OR (((TI quantitative OR AB quantitative) N3 ((TI review* OR AB review*) OR (TI overview* OR AB overview*) OR (TI synthes* OR AB synthes*))) OR ((TI research ...
S25 AND S26

Table S2a. Characteristics of the evidence syntheses examining the relationship between folate status and autoimmune diseases

First author (year)	Synthesis type	Study population	Folate exposure	Outcome	No. of studies (Design)	Total (Case)	Country	Comparators
Zhu Y et al. (2011)	MA	Individuals with MS and healthy controls	Serum folate	MS	6 (CC)	638 (324)	Netherlands, Sweden, Italy, Turkey	Case vs control
Deminice et al. (2015)	MA	Individuals with HIV under antiretroviral therapy and healthy controls	Plasma folate	HIV infection	3 CS, 1 PC, 1 CC	505 (255)	Spain, Italy, Brazil, Argentina	High vs low
Bagur et al. (2017)	SR	NR	Dietary intake	MS	1 (RCT)	37	Iran	High vs low
			Serum folate	MS	1 (RCT)	75	Iran	High vs low
Dardiotis et al. (2017)	MA	Patients with MS and healthy controls (mean age 30-38.5 years)	Plasma/serum folate	MS	9 (CC)	1,340 (655)	Netherlands, Italy, Sweden, Turkey, Iran	Case vs control
Thompson et al. (2017)	SR	Individuals with alopecia areata and healthy controls	Serum, RBC	Alopecia areata	3 (CC)	269 (147)	Iran, Turkey	Case vs control
Fritz et al. (2019)	SR	Children with or without IBD	RBC folate	Crohn's disease<br>Ulcerative colitis<br>Indeterminate colitis	4 prospective, 4 retrospective, 1 NR	303 (NR)<br>590 (NR)<br>4 (NR)	NR	High vs low
Pan et al. (2017)	MA	Individuals with IBD	Serum folate	IBD	11 (CC)	2,522 (1,062)	France, Netherlands, Spain, Greece, US, Japan, China, Turkey, Tunisia	Case vs control
	MA	Individuals with CD	Serum folate	Crohn's disease	6 (CC)	614 (247)	France, Netherlands, Greece, US, Turkey, Tunisia	Case vs control
	MA	Individuals with UC	Serum folate	Ulcerative colitis	7 (CC)	1,831 (680)	Netherlands, Greece, China, US, Turkey	Case vs control
Pan et al. (2019)	MA	Individuals with MS and healthy controls	Plasma folate	MS	6 (CC)	608 (304)	China	Case vs control
	MA		Plasma folate	MS relapse	6 (CC)	643 (344)	Italy, Turkey, China	Case vs control
			Plasma folate	MS remission	3 (CC)	630 (354)	Italy, Turkey, Iran	Case vs control
Tsai et al. (2019)a	MA	Individuals with vitiligo and healthy controls	Serum folate	Vitiligo	9 (CC)	1,050 (672)	NR	Case vs control
	MA	- Progressive vitiligo vs healthy controls	Serum folate	Progressive vitiligo	4 (CC)	363 (181)	NR	Case vs control

	MA	- Stable vitiligo vs healthy controls	Serum folate	Stable vitiligo	4 (CC)	375 (193)	NR	Case vs control
Tsai et al. (2019)b	MA	Individuals with psoriasis and healthy controls	Serum folate	Psoriasis	14 (CC)	1,645 (740)	UK, Norway, Italy, Austria, Spain, Israel, Turkey, Iran, Malaysia	Case vs control
	MA	- excluding individuals with psoriatic arthritis	Serum folate	Psoriasis	10 (CC)	1,014 (517)	Italy, Austria, Turkey, Malaysia	Case vs control
	MA	- individuals with no exposure to MTX	Serum folate	Psoriasis	6 (CC)	769 (393)	Italy, Austria, UK, Turkey	Case vs control
Li X et al. (2020)	MA	Individuals with MS and healthy controls	Serum folate	MS	13 (CC)	2,161 (1,132)	Sweden, Netherlands, Italy, US, Turkey, Iran, Egypt, South Africa, China	Case vs control
Tsai et al. (2021)	MA	Individuals with SLE and healthy controls	Serum folate	SLE	7 (CC)	1,166 (532)	Italy, Poland, Greece, Kuwait, India, Brazil	Case vs control
	MA	Adults with SLE and healthy controls	Serum folate	SLE	4 (CC)	452 (302)	Kuwait, Poland, Italy, Greece	Case vs control
Kirsty et al. (2022)	MA	Individuals with MS and healthy controls	Plasma folate	MS	5 (CC)	587 (329)	Italy, Egypt, Turkey, Iran, China	Case vs control

ARR: absolute risk reduction; CC: case-control study; CD: Crohn's disease; CS: cross-sectional study; FA: folic acid; MA: meta-analysis; MD: mean difference; MS: multiple sclerosis; MTX: methotrexate; PC: prospective cohort; RA: rheumatoid arthritis; RCT: randomized controlled trial; RR: relative risk; SLE: systemic lupus erythematosus; SMD: standardized mean difference; SR: systematic review; UC: ulcerative colitis; WMD: weighted mean difference

Table S2b. Characteristics of the evidence syntheses examining the relationship between folate status and skeletal outcomes

First author (year)	Synthesis type	Study population	Folate exposure	Outcome	No. of studies (Design)	Total (Case)	Country	Comparators
Herrmann et al. (2007)	SR	Older individuals	Plasma folate	Fracture	4 (NR)	2,174 (NR)	NR	High vs low
			Plasma folate	BMD	15 (NR)	9,255 (NR)		
Van Wijngaarden et al. (2013)	SR	Older individuals	Plasma folate	Fracture	3 (PC)	6,423 (NR)	Norway, Italy, US	High vs low
		Older women	Plasma/serum folate	BMD	11 (CS), 1 (PC)	9,355 (NR)	UK, Italy, Norway, US, Turkey, Morocco, Croatia, Iran, Slovakia	High vs low
	MA	Older women	Plasma/serum folate	BMD, femoral neck<br>BMD, lumbar spine<br>BMD, total hip	4 (CS)<br>4 (CS)<br>5 (CS)	1,037 (NR)<br>1,037 (NR)<br>6,366 (NR)	Norway, Italy, Slovakia, Croatia, Morocco	High vs low
Zhang H et al. (2014)	MA	Women with osteoporosis and healthy controls	Serum folate	BMD	6 (CC)	649 (288)	UK Italy, Turkey, Morocco, China	Case vs control
Zhou Q et al. (2016)	MA	Postmenopausal women	Plasma/serum folate	Postmenopausal osteoporosis	6 (CS)	732 (325)	UK, Italy, Morocco, Turkey	Case vs control
He et al. (2021)	MA	Older individuals with severe folate deficiency	Plasma folate	Fracture	5 (PC)	7,835 (NR)	Norway, Sweden, Italy, US, Japan	High vs low
		Older individuals with low folate status	Plasma folate	Fracture	3 (PC)	6,470 (NR)		
Zhao F et al. (2021)	MA	Postmenopausal women	Plasma/serum folate	Postmenopausal osteoporosis	9 (CS)	2,793 (735)	UK, Italy, US, Turkey, Morocco, India	Case vs control

ARR: absolute risk reduction; CC: case-control study; CD: Crohn's disease; CS: cross-sectional study; FA: folic acid; MA: meta-analysis; MD: mean difference; MS: multiple sclerosis; MTX: methotrexate; PC: prospective cohort; RA: rheumatoid arthritis; RCT: randomized controlled trial; RR: relative risk; SLE: systemic lupus erythematosus; SMD: standardized mean difference; SR: systematic review; UC: ulcerative colitis; WMD: weighted mean difference

Table S3a. Summary of the meta-analyses reporting the association of folate status with the risk of autoimmune disease

Author (year)	Outcome	Study population/subgroup	No. studies (design)	Dose, duration, follow-up (mean, range)	No. total (case)	Comparator	Summary effect	I^2	P_{Egger}
**Multiple sclerosis – Plasma/serum folate**									
Zhu Y (2011)	MS	Individuals with MS and healthy controls	6 (CC)	NA	638 (324)	Case vs control	SMD= -0.08 (-0.25, 0.09)	p=0.34	p=0.01
Dardiotis (2017)	MS	Individuals with MS and healthy controls	9 (CC)	NA	1,340 (655)	Case vs control	SMD= -0.08 (-0.24, 0.08)	42% (p=0.09)	No¶
Li X (2020)	MS	Individuals with MS and healthy controls	13 (CC)	NA	1,161 (1,132)	Case vs control	SMD= 0.07 (-0.14, 0.28)	81%	p=0.26
Kirsty (2022)	MS	Individuals with MS and healthy controls	5 (CC)	NA	587 (329)	Case vs control	WMD= 0.00 (-0.01, 0.01)	0% (p=0.31)	NR
Pan (2019)	MS	Chinese individuals with MS and healthy controls	6 (CC)	NA	608 (304)	Case vs control	SMD= -0.12 (-0.29, 0.04)	45.3% (p=0.10)	NR
Pan (2019)	MS, relapsing	Individuals with MS and healthy controls	6 (CC)	NA	643 (344)	Case vs control	SMD= -0.14 (-0.42, 0.06)	53.6% (p=0.06)	NR
Pan (2019)	MS, remitting	Individuals with MS and healthy controls	3 (CC)	NA	630 (354)	Case vs control	SMD= -0.05 (-0.51, 0.40)	76.0% (p=0.02)	NR
**Inflammatory Bowel Disease –Serum folate**									
Pan (2017)	IBD	Individuals with IBD and healthy controls	11 (CC)	NA	2,522 (1,062)	Case vs control	**SMD= -0.46 ng/mL (-0.64, -0.27)**	74% (p=0.000)	NR
		- Asia	5 (CC)	NA	1,609 (662)	Case vs control	**SMD= -0.65 ng/mL (-0.86, -0.44)**	64.3% (p=0.02)	NR
		- Europe	4 (CC)	NA	595 (250)	Case vs control	**SMD= -0.44 ng/mL (-0.62, -0.26)**	3.1% (p=0.40)	NR
**Crohn's Disease – Serum folate**									
Pan (2017)	CD	Individuals with CD and healthy controls	6 (CC)	NA	614 (247)	Case vs control	SMD= -0.30 ng/mL (-0.63, 0.04)	79% (p=0.08)	NR
**Ulcerative Colitis – Serum folate**									
Pan (2017)	UC	Individuals with UC and healthy controls	7 (CC)	NA	1,831 (680)	Case vs control	**SMD= -0.50 ng/mL (-0.71, -0.28)**	63% (p=0.00)	NR
**Psoriasis – Serum folate**									
Tsai (2019b)	Psoriasis	Individuals with psoriasis and healthy controls	14 (CC)	NA	1,645 (740)	Case vs control	**SMD= -0.94 (-1.49, -0.40)**	95.6% (p=0.001)	NR
	Psoriasis	- Excluding individuals with psoriatic arthritis	10 (CC)	NA	1,014 (517)	Case vs control	**SMD= -1.24 (-2.05, -0.43)**	96.7% (p=0.003)	NR
	Psoriasis	- Individuals with no exposure to MTX	6 (CC)	NA	769 (393)	Case vs control	**SMD= -2.13 (-3.29, -0.97)**	97.4% (p<0.001)	NR
**HIV – Plasma folate**									
Deminice (2015)	HIV	Individuals with HIV under antiretroviral therapy and healthy controls	3 CS, 1 PC, 1 CC	NA	505 (255)	Case vs control	**WMD= -2.74 ng/mL (-5.18, -0.29)**	97% (p<0.00001)	NR

**Vitiligo – Serum folate**									
Tsai (2019a)	Vitiligo	Individuals with vitiligo and healthy controls	9 (CC)	NA	1,050 (672)	Case vs control	SMD= -0.24 ng/mL (-0.59, 0.11)	85.5% (p=0.18)	NR
	Vitiligo, progressive	- Progressive vitiligo vs healthy controls	4 (CC)	NA	363 (181)	Case vs control	SMD= -0.40 ng/mL (-1.55, 0.75)	95.7% (p=0.50)	NR
	Vitiligo, stable	- Stable vitiligo vs healthy controls	4 (CC)	NA	375 (193)	Case vs control	SMD= -0.44 ng/mL (-1.41, 0.52)	94.3% (p=0.37)	NR
**Systemic Lupus Erythematosus – Serum folate**									
Tsai (2021)	SLE	Individuals with SLE and healthy controls	7 (CC)	NA	1,166 (532)	Case vs control	SMD= -0.28 (-0.67, 0.12)	86.7% (p=0.18)	NR
	SLE	- Adults	4 (CC)	NA	452 (302)	Case vs control	SMD= -0.36 (-1.15, 0.44)	92.7% (p=0.38)	NR

⁋ as reported by the authors

ARR: absolute risk reduction; CC: case-control study; CD: Crohn's disease; CS: cross-sectional study; FA: folic acid; MA: meta-analysis; MD: mean difference; MS: multiple sclerosis; MTX: methotrexate; PC: prospective cohort; RA: rheumatoid arthritis; RCT: randomized controlled trial; RR: relative risk; SLE: systemic lupus erythematosus; SMD: standardized mean difference; SR: systematic review; UC: ulcerative colitis; WMD: weighted mean difference

Table S3b. Summary of the meta-analyses reporting the association of folate status with the risk of skeletal outcomes

Author (year)	Outcome	Study population/subgroup	No. studies (design)	Dose, duration, follow-up (mean, range)	No. total (case)	Comparator	Summary effect ()	I^2	P_{Egger}
**Fracture – Plasma folate**									
He (2021)	Fracture	Older individuals, severe folate deficiency	5 (PC)	NA	7,835 (NR)	High vs low	**HR=1.46 (1.06, 2.02)**	42.3% (p=0.14)	NR
	Fracture	Older individuals, low folate status	3 (PC)	NA	6,470 (NR)	High vs low	HR=0.79 (0.56, 1.12)	0% (p=0.52)	NR
**Bone Mineral Density/Osteoporosis – Plasma/serum folate**									
Van Wijngaarden (2013)	BMD, femoral neck	Older women	4 (CS)	NA	1,037 (NR)		β=0.00 nmol/L (-0.03, 0.03)	0% (p=0.88)	NR
	BMD, lumbar spine		4 (CS)	NA	1,037 (NR)		β=0.01 nmol/L (0.00, 0.01)	0% (p=0.77)	NR
	BMD, total hip		5 (CS)	NA	6,366 (NR)		β=0.00 nmol/L (0.00, 0.01)	78.5% (p=0.0003)	NR
Zhang H (2014)	Osteoporosis	Women with osteoporosis and healthy controls	6 (CC)	NA	649 (288)	Case vs control	MD= -1.54 (-3.33, 0.25)	94% (p<.00001)	NR
Zhou (2016)	Postmenopausal osteoporosis	Postmenopausal women	6 (CS)	NA	732 (325)	Case vs control	**SMD=-1.18 (-2.04, -0.31)**	96.4% (p<.0001)	NR
Zhao (2021)	Postmenopausal osteoporosis	Postmenopausal women	9 (CS)	NA	2,793 (735)	Case vs control	**SMD=-1.16 (-2.23, -0.08)**	98.9% (p=0.000)	No⁋
		- Asia	4 (CS)	NA	350 (172)	Case vs control	SMD=-0.28 (-0.74, 0.19)	76.2%	NR
		- Europe	3 (CS)	NA	535 (197)	Case vs control	**SMD=-1.98 (-3.70, -0.25)**	98.4%	NR
		- Serum samples only	8 (CS)	NA	NR (NR)	Case vs control	**SMD=-1.27 (-2.45, -0.10)**	NR	NR

⁋ as reported by the authors

BMD: bone mineral density; CC: case-control study; CS: cross-sectional study; FA: folic acid; HR: hazard ratio; MA: meta-analysis; MD: mean difference; PC: prospective cohort; RCT: randomized controlled trial; RR: relative risk; SLE: systemic lupus erythematosus; SMD: standardized mean difference

Table S4a. Risk of bias assessment of the syntheses examining the association between folate status and autoimmune diseases

	Domain 1: Study eligibility criteria	Domain 2: Identification and selection of studies	Domain 3: Data collection and study appraisal	Domain 4: Synthesis and findings	Risk of Bias in the Review
Bagur M (2017)	⊕	⊖	⊗	⊗	⊗
Dardiotis E (2017)	⊕	⊗	⊕	⊕	⊗
Deminice R (2015)	⊕	⊗	⊗	⊕	⊗
Fritz J (2019)	⊕	⊕	⊕	⊕	⊕
Kirsty CW (2022)	⊕	⊕	⊕	⊕	⊕
Li X (2020)	⊗	⊗	⊗	⊖	⊗
Pan L (2019)	⊕	⊗	⊕	⊕	⊗
Pan Y (2017)	⊕	⊗	⊕	⊕	⊗
Thompson J (2017)	⊗	⊗	⊗	⊗	⊗
Tsai TY (2019)	⊕	⊕	⊕	⊕	⊕
Tsai TY (2019)	⊕	⊕	⊕	⊕	⊕
Tsai TY (2021)	⊕	⊗	⊕	⊕	⊗
Zhu Y (2011)	⊕	⊗	⊕	⊕	⊗
High	2	8	4	2	9
Low	11	4	9	10	4
Unclear	0	1	0	1	0

Table S4b. Risk of bias assessment of the syntheses examining the association between folate status and skeletal outcomes

	Domain 1: Study eligibility criteria	Domain 2: Identification and selection of studies	Domain 3: Data collection and study appraisal	Domain 4: Synthesis and findings	Risk of Bias in the Review
He T (2021)	⊕	⊕	⊕	⊕	⊕
Herrmann M (2007)	⊖	⊗	⊗	⊗	⊗
van Wijngaarden JP (2013)	⊕	⊕	⊕	⊗	⊗
Zhang H (2014)	⊕	⊕	⊕	⊕	⊕
Zhao F (2021)	⊕	⊕	⊕	⊕	⊕
Zhou Q (2016)	⊕	⊗	⊗	⊕	⊗
High	0	2	2	2	3
Low	5	4	4	4	3
Unclear	1	0	0	0	0
